# Sarcomatoid Carcinoma Arising in the Gallbladder of a 58-Year-Old Female: Case Report

**DOI:** 10.1155/crhe/5172834

**Published:** 2025-06-26

**Authors:** Ahmed A. Ahmed, Yan Gao, Rossana Kazemimood

**Affiliations:** Department of Pathology and Laboratory Medicine, McGovern Medical School, The University of Texas Health Science Center, Houston, Texas, USA

## Abstract

Sarcomatoid carcinoma of the gallbladder is a rare malignancy with no specific clinical manifestation. It is characterized by the presence of malignant epithelial and mesenchymal components intermingled with each other. This disease usually presents at an advanced stage, and its final diagnosis needs to be confirmed by histopathological and immunohistochemical examination. Sarcomatoid carcinoma is characterized by early metastasis through lymphatics, rapid progression, a high recurrence rate, and a worse prognosis than usual gallbladder adenocarcinoma. Herein, we report the case of a 58-year-old female patient, who underwent laparoscopic cholecystectomy, with sarcomatoid carcinoma of the gallbladder. Histologically, the epithelial component of the tumor was composed of adenocarcinoma, and the mesenchymal component was composed of spindle cell sarcoma and chondrosarcoma. The tumor was identified as invading the perimuscular connective tissue on the hepatic side without involvement of the liver. The prognosis of sarcomatoid carcinoma of the gallbladder remains poor despite surgical resection of the gallbladder. The patient passed away in less than 1 year after the procedure despite chemotherapy due to cancer progression.

## 1. Introduction

Sarcomatoid carcinoma of the gallbladder is a notoriously lethal malignancy that originates from mesenchymal stem cells. By morphology and immunohistochemistry, sarcomatoid carcinoma shows both epithelioid and mesenchymal components intermixed with each other. Sarcomatoid carcinomas are rare and can occur in all parts of the body, but they are common in the lungs, upper respiratory tract, prostate, and kidneys. Sarcomatoid carcinoma of the gallbladder is rarely observed [1]. Only a few cases of sarcomatoid carcinoma of the gallbladder have been reported, and the clinical presentations and treatment managements of this disease are still in the exploratory stage.

## 2. Case Report

A 58-year-old female with a past medical history of chronic cholecystitis, hypertension, and diabetes mellitus presented to our institution with intermittent, sharp pain in the right upper quadrant and epigastric region, occasionally radiating to the right mid-back. These symptoms had been ongoing for several weeks. She also reported multiple episodes of bilious vomiting. Approximately 6 months prior, she experienced similar pain for over 5 months and underwent an abdominal ultrasound, which revealed cholelithiasis and gallbladder sludge without signs of acute cholecystitis. Based on a recent hepatobiliary iminodiacetic acid (HIDA) scan suggesting acute cholecystitis with cystic duct obstruction, her primary care physician advised her to seek emergency evaluation. In the emergency department, laboratory workup results were within the normal range. Abdominal imaging confirmed the presence of multiple gallstones but showed no evidence of pericholecystic fluid or gallbladder wall thickening. Following surgical evaluation, she underwent a laparoscopic cholecystectomy with pathological assessment of the removed specimen. Preoperatively, tumor marker testing revealed a markedly elevated CA 19-9 level of 1942.20 U/mL (reference range: < 35.00 U/mL), while CEA levels remained within normal limits.

## 3. Results

### 3.1. Macroscopic Features

By gross examination, the specimen consisted of a disrupted gallbladder measuring 10.0 × 4.5 × 3.5 cm with diffusely erythematous and slightly dull serosa. The gallbladder neck was slightly distended, appeared firm, and measured 2.5 × 2.5 × 2.5 cm. It was opened to reveal a tan-red, diffusely denuded mucosa with multiple tan-yellow bosselated friable calculi. Additionally, single calculus lodged within the neck of the specimen which completely obstructed the cystic duct. The gallbladder was serially sectioned to reveal tan-white dense cut surfaces with focal areas of hemorrhage. The wall was slightly trabeculated, and a tan-white focal area of fibrous tissue was located toward the fundus on the adventitial side of the specimen. The neck appeared tan-white and fibrous. The wall was approximately 0.1–0.8 cm in thickness. No periductal lymph node was grossly identified. The specimen was submitted entirely for microscopic examination.

### 3.2. Microscopic Features

**Figure 1:**
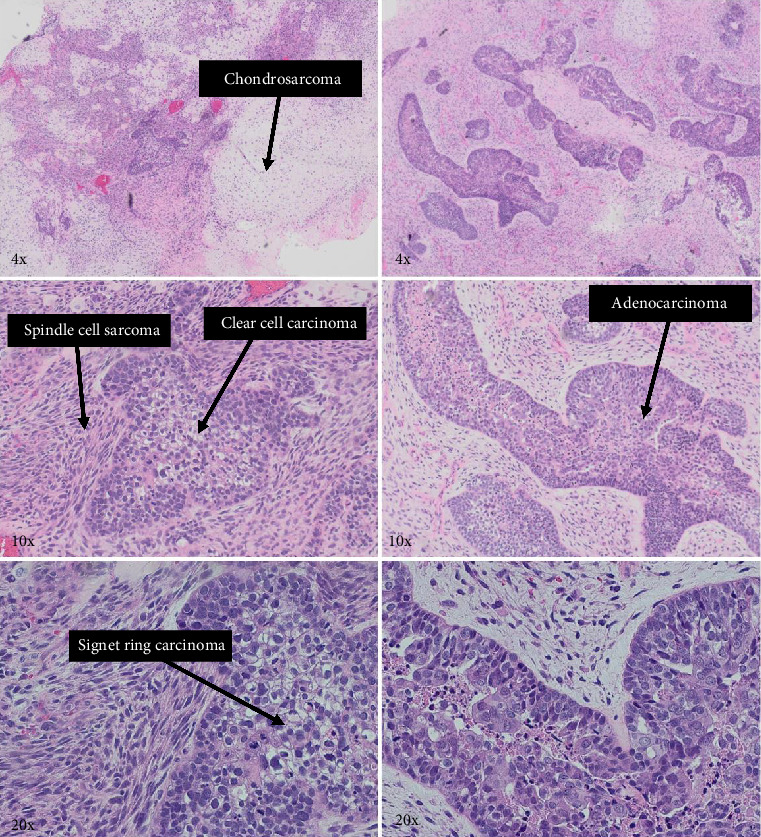
H&E sections showing both components of carcinoma (adenocarcinoma, clear cell carcinoma, and signet ring carcinoma) and sarcoma (spindle cell sarcoma and chondrosarcoma) intermingle with each other.

**Figure 2:**
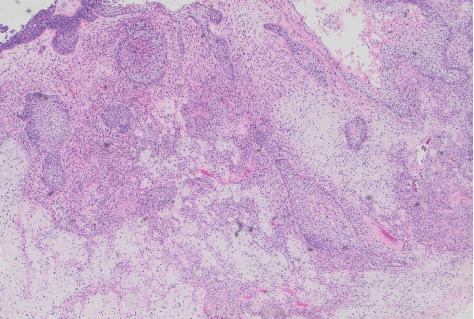
H&E sections showing the deepest invasive portion of tumor extending into the perimuscular connective tissue on the hepatic side.

Histologic examination of the lesion revealed a conventional adenocarcinoma composed of well-formed glands arising in a background of flat dysplasia. Notably, there was a polypoid component measuring 1.5 cm in greatest dimension of carcinosarcoma. This area demonstrated an intimate admixture of both epithelial and mesenchymal elements. The epithelial carcinomatous component included adenocarcinoma, clear cell carcinoma, and signet ring cell carcinoma, while the mesenchymal sarcomatous areas consisted of spindle cell sarcoma and chondrosarcoma. Of notice, histomorphologically, the epithelial elements displayed a combination of glandular structures, anastomosing cords, nests, trabeculae, and sheets of single cells in architecture. Cytologically, many cells showed glycogen-rich clear cytoplasm and/or intracytoplasmic mucin but with more basaloid to squamoid cells at the periphery of epithelial nests (Figure 1). Importantly, the deepest invasive portion of the tumor was composed of sarcomatoid elements, which extended into the perimuscular connective tissue on the hepatic side, without direct liver invasion. The presence of lymphovascular and perineural invasion was also noted. This deep mesenchymal invasion may correlate with the tumor's aggressive clinical course and poor prognosis (Figure 2). The cystic duct and hepatic bed soft tissue margins were free of invasive carcinoma and high-grade intraepithelial neoplasia. The surgical margins were negative, with the closest margin being the hepatic bed soft tissue margin, measuring 1.5 mm.

### 3.3. Immunohistochemical Findings

**Figure 3:**
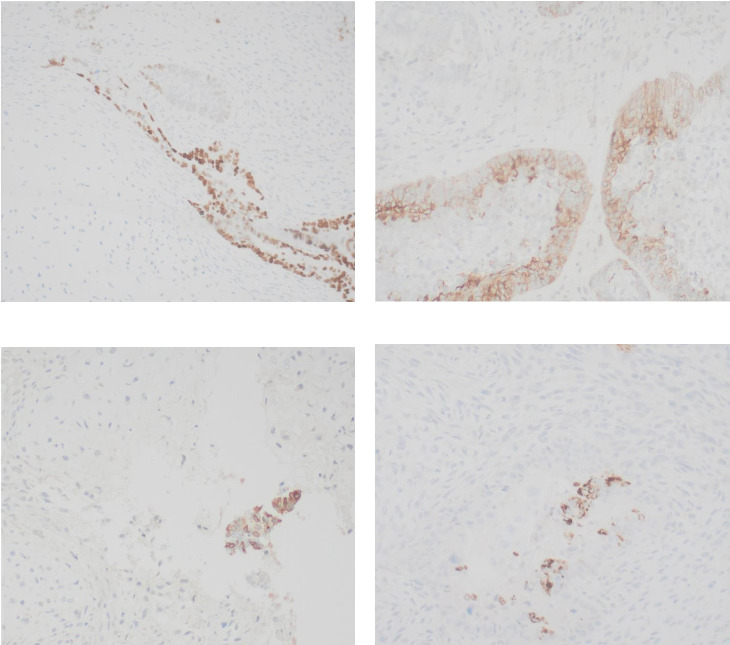
(a) SALL4 (patchy). (b) Glypican-3 (patchy). (c) AFP (focal). (d) Hep-Par1 (rare).

**Figure 4:**
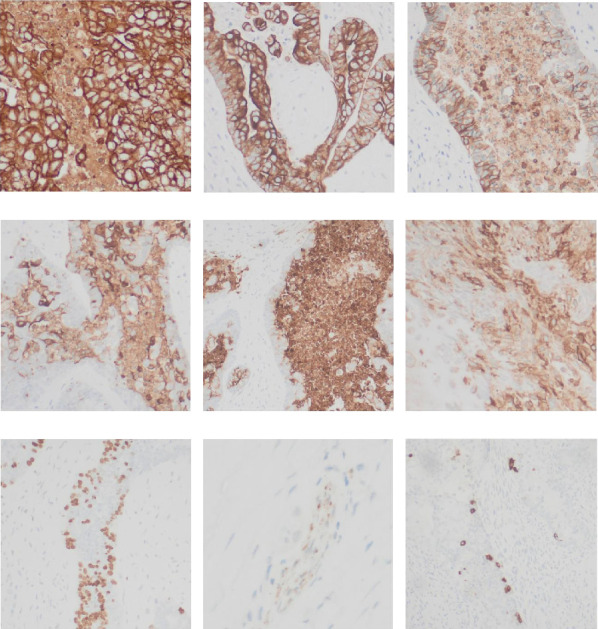
(a) AE1/AE3 (diffuse). (b) CK7. (c) MOC31 (patchy). (d) EMA (patchy). (e) CEA (patchy). (f) CD10 (patchy and variable). (g) p63. (h) Chromogranin (scattered). (i) Synaptophysin (scattered).

Immunohistochemical stains showed that the cells of the carcinoma component were positive for SALL4 (patchy), Glypican-3 (patchy), AFP (focal), and Hep-Par1 (rarely), features of fetal gut-like adenocarcinoma/hepatoid adenocarcinoma (Figure 3). The clear cell carcinoma/signet ring carcinoma components were positive for AE1/AE3 (diffuse), CK7 (positive for adenocarcinoma and signet ring carcinoma), MOC31 (patchy), EMA (patchy), CEA (patchy), CD10 (patchy and variable), p63 (positive in basaloid/squamoid cells), chromogranin, and synaptophysin (scattered positive cells), while negative for CK20, PAX-8, ER, and inhibin (Figure 4). The chondrosarcoma component was positive for S100 and negative for pan-cytokeratin and other markers. The spindle cell sarcoma component was negative for pan-cytokeratin and S100.

A few weeks later, the patient underwent endoscopic retrograde cholangiopancreatography (ERCP) with metal stent placement due to tumor progression involving the liver and peritoneal wall. This was followed by six cycles of adjuvant chemotherapy with cisplatin and gemcitabine. Despite initial treatment, subsequent imaging revealed further metastatic spread of the tumor. As a result, FOLFOX chemotherapy was initiated. Although the patient initially tolerated the regimen, she later developed intractable nausea and vomiting. Her clinical condition progressively declined despite supportive interventions. Unfortunately, she passed away 10 months after the initial diagnosis due to complications from an aspiration event.

## 4. Discussion

The first case of sarcomatoid carcinoma of the gallbladder (previously named gallbladder carcinosarcoma) was described by Landsteiner in 1907 [2]. Carcinomas of the gallbladder are rare, with adenocarcinoma being the most common histological subtype (85%), then anaplastic (5%), squamous cell (4%), adenosquamous (3%), and small cell (1%–3%) subtypes [3]. Sarcomatoid carcinoma of the gallbladder is even more rare, accounting for < 1% of gallbladder carcinomas. Till today, the number of cases diagnosed with this tumor is limited due to the invasive nature and aggressiveness of the disease, and this is why the knowledge and experience regarding sarcomatoid carcinoma of the gallbladder needs more discussion [4].

Sarcomatoid carcinoma of the gallbladder typically consists of mixed malignant epithelial and mesenchymal components. The diagnosis requires the presence and intermixing of both histological components. The epithelial component usually consists of a carcinoma (e.g., adenocarcinoma, squamous cell carcinoma, clear cell, or signet ring carcinoma). The mesenchymal component typically consists of spindle- and fibroblast-like cells, but they are more commonly pleomorphic (including giant cells) and is occasionally accompanied by other heterologous differentiation (i.e., skeletal muscle, bone, and cartilage) [5]. In this case, the epithelial component of the tumor consisted of adenocarcinoma, clear cell, and signet ring carcinoma, while the mesenchymal component consisted of spindle cell sarcoma and chondrosarcoma. Immunohistochemical examination revealed that the epithelial component was positive for pan-cytokeratin, while the chondrosarcoma component was positive for S100.

The overall 5-year survival rate of the gallbladder carcinomas is < 10% due to tumor extreme aggressiveness. Chronic gallbladder stones (cholelithiasis) are considered a major risk factor. Almost 50% of the cases were detected incidentally in routine cholecystectomy specimens due to the absence of any gross abnormalities, and this explains why systematic sampling of gallbladder specimens is crucial to detect incidental carcinomas [6]. Moreover, the real causes of this carcinoma are not fully understood, such as different genetic alterations, and the precise mechanisms of gallbladder carcinogenesis have not been made clear. Two opposing theories have been hypothesized to reveal the origin of these morphologically diverse components. The first theory, the multiclonal origin, considers a sarcomatoid carcinoma as a collision tumor composed of the byproducts of two or more stem cells of separate epithelial and mesenchymal origins. While the second theory, the monoclonal origin, suggests that the elements of carcinoma and sarcoma are derived from a single stem cell (pluripotential stem cell) that afterward develops divergent differentiation along distinct epithelial and mesenchymal pathways [7].

Sarcomatoid carcinoma of the gallbladder can develop at any age. It has a wide age range (40–90 years), and the mean age at diagnosis is during the sixth decade of life [8]. The preoperative diagnosis of this tumor is challenging due to its nonspecific clinical presentation and imaging findings. The final diagnosis requires histopathological confirmation of the presence of intermixed epithelial and mesenchymal components [9]. Representative clinical symptoms are also nonspecific and include right upper abdominal pain with or without masses, decreased appetite, weight loss, fatigue, jaundice, nausea, and vomiting. Furthermore, over two-thirds of the cases present with concurrent gallstones [10, 11]. The usual treatment of choice for this disease is surgery, and no successful treatment has been reported with radiotherapy or chemotherapy [12].

The prognosis of this disease is generally poor despite aggressive surgical resection as most cases usually present with locally advanced disease. This tumor spreads by direct invasion to adjacent organs (e.g., liver) through vessels or lymphatics. Cases presented with extensive metastasis to the peritoneum, pancreas, adrenal glands, diaphragm, and vertebrae have been reported. Metastasis to regional, retroperitoneal, and para-aortic lymph nodes is another possibility. Even when a curable resection is guaranteed, many patients die shortly after the operation because of aggressive recurrence or metastasis [13–15], as in the case presented here. Prolonged survival can only be achieved with proper surgical treatment in cases with a tumor protruding into the lumen and without invasion of the liver or serosa, or lymph node involvement [7].

## 5. Conclusion

Sarcomatoid carcinoma of the gallbladder is an extremely rare tumor, and a differential diagnosis can be missed by treating physicians. It is a disease in females, in their sixth decade of life, which usually presents with abdominal pain as a sole complaint with normal laboratory workup.

The current knowledge and predictions regarding sarcomatoid carcinoma of the gallbladder are based on a limited number of case reports. The precise mechanisms of gallbladder carcinogenesis are still unknown and need more research and discussion. These cases are to be reported to gain knowledge of the distinct characteristics of such notoriously lethal disease.

## Data Availability

The data that support the findings of this study are available from the corresponding author upon reasonable request.
